# Cisplatin-Induced Renal Failure Measured by Glomerular Filtration Rate (GFR) with ^99^mTc-DTPA Scans in Cancer Patients: A Systematic Review and Meta-Analysis

**DOI:** 10.3390/diagnostics14222468

**Published:** 2024-11-05

**Authors:** Mansour M. Alqahtani

**Affiliations:** Department of Radiological Sciences, College of Applied Medical Sciences, Najran University, Najran 61441, Saudi Arabia; mmalqahtane@nu.edu.sa

**Keywords:** cisplatin, anti-cancer, cisplatin-induced renal injury, nephrotoxicity, renal failure

## Abstract

**Background**: Cisplatin is a potent agent commonly used to treat cancer, but its effects pose a significant risk to renal function. Therefore, the present study aimed to evaluate the impact of cisplatin on renal function as measured by glomerular filtration rate (GFR) using diethyltriamine-penta-acetic acid (DTPA) renal scintigraphy. **Methods**: Extensive literature searches were performed using PRISMA guidelines that investigated cisplatin-induced renal failure by measuring GFR with DTPA. Eligible studies were included based on predefined criteria. Data on GFR, serum creatinine levels, and acute kidney injury (AKI) before and after cisplatin therapy were extracted and analyzed. A meta-analysis was performed utilizing RevMan 5.4 to determine the overall effect of cisplatin on GFR before and after treatment. For non-randomized controlled trials (RCTs), quality assessment was performed using the Newcastle–Ottawa Scale, while for RCT, the Cochrane risk of bias tool was utilized. **Results**: Initially, 1003 studies were searched from different databases, including ScienceDirect, PubMed, Scopus, Google Scholar, and The Cochrane Library, and after screening, 8 studies (PubMed, Scopus, and GoogleS cholar) with 489 patients were found eligible for inclusion in the present study. Cisplatin was administrated with varying doses ranging from 20 mg/m^2^ to 114.02 mg/m^2^. The findings underscore the nephrotoxic effects of cisplatin, a widely used chemotherapeutic agent, as demonstrated by the significant decline in GFR observed across multiple treatment cycles, and these findings were also supported by the findings of a meta-analysis that showed a significant (*p* < 0.01) difference between peri- and post-treatment GFR level with 37.06 (95% CI, 10.90–63.23) effect size and 96% heterogeneity. In addition, the included studies were found to be of high quality. **Conclusions**: Cisplatin significantly affects renal function, as evidenced by a decrease in GFR measured with DTPA. The findings underscore the importance of the routine monitoring of GFR to detect early renal injury and guide treatment modification. Future research should focus on strategies to reduce cisplatin-induced toxicity and explore alternative therapies with reduced renal risk.

## 1. Introduction

Cancer is considered one of the most significant global threats, causing one in every sixth deaths [1]. During 2022, 20 million new cases with 9.7 million deaths were reported, while cancer patients alive within 5 years following their cancer diagnosis were 53.5 million, and one in five individuals will develop any type of cancer in their life. Meanwhile, in 2040, cases are expected to reach 28.4 million [2,3]. Despite significant advancements in different treatment approaches, including surgical, immunological, and radiotherapy, which have improved and enhanced the clinical outcomes of cancer management, chemotherapy has its own role to play, and serves as a key therapeutic strategy [4].

Among the methods of chemotherapy, the use of metal-based drugs has gained an important role in cancer therapy, enhancing the treatment [5]. Metal-based drugs include cisplatin, also known as Cis-Diammine-Dichloroplatinum (CDDP), which is a well-known chemotherapeutic agent used for several human cancers, including head and neck, bladder, lung, testicular, and ovarian, and found to be very much effective against germ-cell tumors, carcinomas (the uncontrolled growth of epithelial tissue, characterized by severe pathology extending throughout the entire thickness of the epithelium with the extension of rete pegs into the underlying lamina propria, indicating invasion beyond the epithelial basement membrane; such a local invasion may eventually spread via the lymphs to distant organs like the liver, brain and bones), lymphomas, and sarcomas [6,7]. The most significant advantage of cisplatin is its multidirectional mode of action, with the most significant direction being the damaging of cancer cells’ DNA [8]. However, it is also considered one of the alkylating nephrotoxic drugs; therefore, before starting the therapy with this particular agent, it is necessary to know that renal function is normal [9]. Nephrotoxicity is the cisplatin dose-limiting factor, and this phenomenon is characterized by reduced renal function; its effects can be seen years after treatment has ceased [10]. Cisplatin-induced renal failure is a multifaceted condition resulting from the accumulation of cisplatin in renal tubular cells [11] and interference with the functioning of numerous organelles, including lysosomes, mitochondria, endoplasmic reticulum, nuclei, and cell membranes [12], leading to cellular injury through the generation of reactive oxygen species (ROS), oxidative stress, vascular injury in the kidneys, inflammation, and apoptosis [13,14]. Meanwhile, 20–30% of cancer patients develop acute kidney injury (AKI); when patients are treated with cisplatin, improvements are observed, and patients who develop AKI have an increased mortality risk and are more likely to develop chronic kidney injury [15,16]. Therefore, understanding the diagnosis biomarkers, such as Glomerular Filtration Rate (GFR) and extent of cisplatin-induced renal toxicity, is essential for developing strategies to mitigate its adverse effects and improve patient outcomes.

As estimated, GFR is very much associated with the progression of renal disease, and it also reflects many of the physiological functions of the kidney [17]. The accurate measurement of GFR is paramount in assessing renal function, particularly in patients at risk of nephrotoxicity due to cisplatin [18], which allows for the early determination of cisplatin-induced nephrotoxicity [19]. Different traditional methods, such as plasma kidney injury molecules-1, urinary creatinine clearance (uCrCl), serum creatinine, and serum electrolytic alterations, have been used for the measurement of GFR [20,21]. In addition, multiple logistic regression analysis indicates that cystatin C-based GFR measurement provides a more accurate reflection of deterioration from AKI. Cystatin C facilitates faster healing and may help improve clinical outcomes [22]. However, these methods might often be influenced by factors like age, gender, and muscle mass, leading to inaccurate assessments of cancer patients.

GFR can be accurately measured from the clearance rate of a tracer activity, commonly known as ^99^mTc DTPA, from the plasma, which is considered a precise and gold standard method [23]. Nevertheless, evaluating GFR using camera-based ^99^mTc DTPA renal scintigraphy is a noninvasive, efficient method, and is less time consuming [23]. This approach also allows for the assessment of individual renal function, unlike other methods that only evaluate global renal performance. However, scintigraphy has limitations, including the use of radioactive isotopes, the requirement for a specialized gamma camera, and the need for expertise in procedure evaluation [24,25]. This technique involves the intravenous injection of radiolabeled ^99^mTc tracer, cleared by glomeruli and excreted by the kidney [26]. The rates of radiotracer clearance from the blood and kidney uptake are measured with gamma cameras to accurately quantify GFR [27].

Despite the recognized nephrotoxic effects of cisplatin, there is a paucity of comprehensive analyses synthesizing the available evidence on the magnitude and mechanisms of cisplatin-induced renal failure, as measured by GFR using ^99^mTc DTPA. Given the importance of accurately monitoring renal function in cancer patients receiving cisplatin, a systematic review and meta-analysis are warranted to consolidate the current understanding, identify gaps in knowledge, and guide clinical practice. This systematic review and meta-analysis aimed to evaluate the impact of cisplatin on renal function, particularly focusing on GFR measurements obtained through ^99^mTc DTPA renal scintigraphy in cancer patients.

## 2. Materials and Methods

### 2.1. Study Design

The present study protocol was developed in accordance with the guidelines provided by Preferred Reporting for Systematic Reviews and Meta-analysis (PRISMA) [28].

### 2.2. Search Strategy

An advanced literature search was performed using different databases, including ScienceDirect, PubMed, Scopus, Google Scholar, and The Cochrane Library, up until 2024. Different keywords such as “cisplatin” OR “metal-based drug” OR “platinol” AND “kidney failure” OR “kidney damage” OR “renal insufficiency” OR “renal failure” OR “kidney dysfunction” OR “nephrotoxicity” AND “glomerular filtration rate” OR “GFR” OR “estimated GFR” OR “eGFR” AND “diethyl-triamine-penta-acetic acid” OR “DTPA” OR “Tc99m DTPA” OR “nuclear medicine” OR “renal scintigraphy” OR “creatinine clearance” AND “cancer” OR “oncology” OR “neoplasms” OR “malignancy” OR “tumours” OR “lung cancer” OR “breast cancer” OR “head and neck cancer” OR “bladder cancer” OR “ovarian cancer” brain tumour”, and combinations of these keywords, were also utilized (see Appendix A Table A1).

### 2.3. Eligibility Criteria

#### 2.3.1. Inclusion Criteria

Certain inclusion criteria were set for studies to be included in the present study, as follows: Studies that reported cancer patients treated with cisplatin regardless of dosage or duration, as chemotherapy. Studies with changes in GFR measured using ^99^mTc DTPA renal scintigraphy. Studies reporting GFR measurements at multiple time points during and after cisplatin chemotherapy. Randomized controlled trials (RCTs), non-RCTs (cohort studies, retrospective, case–control studies, prospective, observational), and studies published in English without publication time restriction.

#### 2.3.2. Exclusion Criteria

Similarly, different exclusion criteria were also set for the studies, as follows: Studies that reported non-cancer patients treated with cisplatin or performed on other types of interventions or with incomplete information. Studies that included cancer patients with pre-existing kidney diseases or patients treated with combined chemotherapy (cisplatin and any other drug). Reviews, letters, editorials, commentary, opinion papers, conference papers, animal studies or case studies with single cases, as well as non-English studies, were excluded.

### 2.4. Study Selection

The study selection process was critically evaluated by two independent reviewers. After the collection of research articles from different databases, the reviewers examined the titles and abstracts of each research article, and all duplicate searched research articles were excluded using Endnote X9 reference software. For final selection, a full-text screening was performed, and search articles were selected following the eligibility criteria. Thus, the information was included when it met inclusion criteria.

### 2.5. Data Extraction

Predefined data variables were extracted using a Microsoft Excel sheet.

Study characteristics: study ID, country, study design.

Participant’s characteristics: sample size, age, gender, type of cancer.

Intervention characteristics: cisplatin dosage.

Outcomes: GFR before treatment, GFR after 1st cycle, 2nd cycle, 3rd cycle and so on, serum creatinine (baseline and after treatment), AKI incidence, and conclusion.

### 2.6. Quality Assessment

The methodological quality of RCTs was evaluated using the Cochrane Risk of Bias (RoB) tool utilizing the web-based application (Robvis) [29]. Studies were characterized in each domain as either low, high, or having some concerns. Outcomes were reported in the form of visualization judgments associated with each RoB item and presented as percentages. The Newcastle–Ottawa Scale was utilized to evaluate the quality of the included studies with the help of two reviewers. Generally, studies were classified as “high-quality” based on the following criteria: (1) the explanation of study protocol, (2) clearly defined eligibility criteria, (3) comprehensive information regarding treatment-related outcomes, and (4) sufficient clinical and radiologic follow-up. Consequently, each study received a star rating ranging from 0 to 9. Studies awarded 6 or more stars were deemed to be of high quality.

### 2.7. Statistical Analysis

Tables and graphs to reflect qualitative data were generated using a Microsoft Excel spreadsheet, while RevMan 5.4 was used for the meta-analysis to calculate the pooled change in GFR overtime (peri and post treatment). The random effects model was used, with the significance level set at 0.01.

## 3. Results

### 3.1. Literature Search

**Figure 1:**
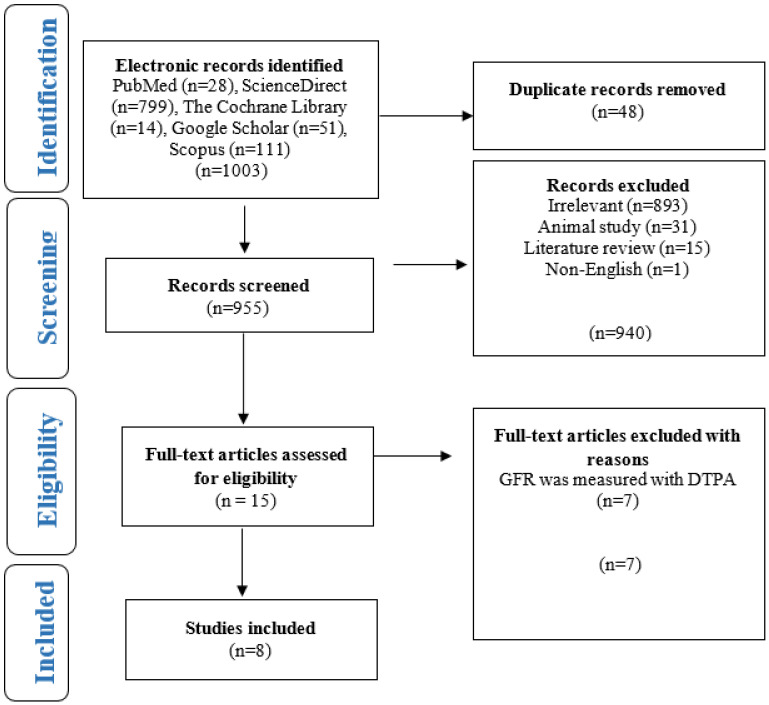
PRISMA flow chart for the selection of studies.

During a literature search in the first phase of identification, 1003 studies were identified and retrieved. During the screening phase, 48 duplicate studies were identified and removed, leaving 955 to be screened for relevance. We then further screened the title and abstract of each study, and 940 studies were identified and removed due to the irrelevancy, including studies performed on animals, reviews, and non-English studies. In the eligibility phase, 15 studies were evaluated through full-text assessment. Finally, in the eligibility phase, seven studies were excluded due to the different reasons listed in Figure 1, leaving eight studies included in the qualitative synthesis and three studies for quantitative synthesis.

### 3.2. General Characteristics

India was identified as the leading country in terms of number of publications, and reported three studies [9,30,31], followed by Pakistan [32,33], while one study each was reported by Denmark [34], Turkey [35], and Malaysia [36]. Most of the studies followed retrospective [31,34,35], prospective [9,36], descriptive [32,33], or RCT designs [30]. Overall, 489 patients were included in the selected studies with a minimum of 9 patients [34] and maximum of 200 patients [31]. In terms of gender, males numbered higher than females, which may be due to the cancer type, as most of the patients were found with testicular cancer, as indicated in Table 1. Furthermore, cisplatin was administrated at varying dose ranging from 20 mg/m^2^ to 114.02 mg/m^2^ [32,33,34]. Total numbers of cycles also varied, and ranged from one to six [9,30,32,33,34]. In addition, ^99^mTc DTPA was administrated IV at different doses, as described in Table 1.

### 3.3. Impact of Cisplatin on GFR and Serum Creatinine

Table 2 summarizes the effects of cisplatin on kidney function, particularly GFR and serum creatinine levels. A significant decline in GFR across multiple cycles was observed, indicating early nephrotoxicity due to cisplatin [30,32,33]. Likewise, there was a decrease in GFR immediately and seven months after chemotherapy, with serum creatinine levels rising temporarily [35]. Furthermore, a higher baseline renal insufficiency detection rate using dGFR compared to serum creatinine levels was seen [9]. Moreover, nephrotoxicity was considered a significant side effect of platinum-based drugs, with substantial GFR reduction observed in early treatment cycles [31]. Meanwhile, no correlation was observed between chronic nephrotoxicity and acute kidney function changes [34].

### 3.4. Meta-Analysis

**Figure 2:**
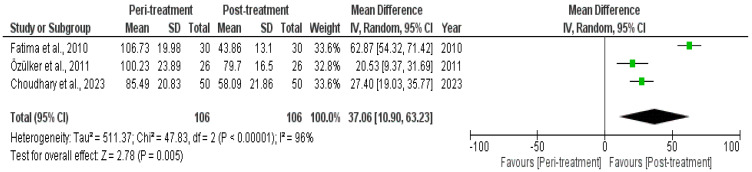
Forest plot showing peri- and post-treatment GFR measured with ^99^mTc DTPA scans.

Due to the unavailability of the required data, only three studies were included in the meta-analysis showing the impact of cisplatin on GFR level. A significant (*p* < 0.01) difference was observed between peri- and post-treatment GFR level, with a 37.06 (95% CI, 10.90–63.23) effect size and 96% heterogeneity (Figure 2).

### 3.5. Quality Assessment

All the studies were determined to be of high quality, as each received a rating of ≥6 quality points according to the Newcastle–Ottawa Scale. This assessment reflects the robustness of the methodologies employed across the studies, including predefined study protocols, clearly defined characteristics for patient selection, detailed reporting on treatment-related outcomes, and adequate clinical outcomes with complete follow-up (Table 3), while one RCT was found with some concern of randomization [30].

## 4. Discussion

This systematic review and meta-analysis focused on evaluating cisplatin-induced renal failure in cancer patients by measuring GFR with ^99^mTc DTPA, and provides important insights into the toxicity of chemotherapy, in which it has found widespread use. Understanding the extent of renal failure determined by GFR measurements quantified by ^99^mTc DTPA measurements is essential to the better management of patients and the development of strategies to reduce this risk.

The present study’s findings underscore the nephrotoxic effects of cisplatin, a widely used chemotherapeutic agent, as demonstrated by the significant decline in GFR observed across multiple treatment cycles quantified with DPTA (Table 2). The ^99^mTc DTPA renal scan is highly effective for estimating GFR, showing excellent correlation with the gold standard techniques used for this purpose [37,38]. Likewise, in another study, the largest proportion of patients showed normal GFR as assessed by DTPA kidney scan results, numbering 83 (90.2%). Further, twenty-four-hour urinary creatinine clearance revealed 59 (64.1%) patients with normal GFR compared with 44 (47.8%) for the chronic kidney disease epidemiology (CKD-EPI) regimen, 39 (42.4%) for the modification of diet in renal disease (MDRD) formula, and 40 (43.5%) individuals were identified with normal GFR for the Cockcroft–Gault (CG) formula [39].

Meanwhile, cisplatin’s nephrotoxicity primarily stems from its accumulation in the kidneys, where it induces inflammation, oxidative stress, and direct tubular cell injury [40,41,42]. This leads to both acute and chronic impairments in kidney function, often manifesting as reduced GFR. The significant difference (*p* < 0.01) observed between peri- and post-treatment GFR levels in the present study’s meta-analysis (Figure 2) further supports the idea that the nephrotoxic effects of cisplatin are both immediate and sustained. The mechanism involves damage to the renal tubular cells and vascular endothelium, which impairs the kidney’s ability to filter blood efficiently, leading to a measurable decline in GFR [43]. Furthermore, it also has intracellular effects, like regulating genes and the activation of mitogen-activated protein kinases, including fibrogenesis and apoptosis [44]. Additionally, factors such as cumulative dose, individual patient susceptibility, and the concurrent use of nephron-protective agents or interventions can influence the extent of renal impairment [45]. Importantly, the decline in GFR serves as a more sensitive and early indicator of renal damage compared to serum creatinine levels, which may not rise until significant nephron loss has occurred [46]. Our findings are in agreement with the findings of a retrospective chart review, and we observed 34.2% AKI incidence in 82 cisplatin-treated head and neck cancer patients, with a mean decrease of 12.57 mL/min/1.73 m^2^ in GFR observed, while after 1 year and the last follow-up, the GFR decreased to <60 mL/min/1.73 m^2^ [47]. Similarly, another retrospective chart review also observed a mean decrease of 12.32 mL/min/1.73 m in GFR when head and neck cancer patients were treated with cisplatin [48]. Moreover, a meta-analysis also observed similar findings, as the pooled reduced GFR prevalence was 29% (95% CI, 0.00–58%), keeping the threshold level at 90 mL/min/1.73 m^2^ [49].

Meanwhile, when cisplatin was combined with metformin and compared with patients treated with only the cisplatin group, the combination group patients showed a lower incidence of nephrotoxicity; however, that difference was statistically non-significant (*p* < 0.05) [50]. This may be due to the protective role of metformin against cisplatin-induced toxicity, primarily undertaken through its antioxidant and anti-inflammatory properties by activating the AMP-activated protein kinase (AMPK) pathway, which reduces oxidative stress and inhibits inflammatory cytokines [51].

Furthermore, another study analyzed the medical records of gastric cancer patients undergoing cisplatin. The patients were divided into two groups: those receiving a conventional hydration regimen and those receiving a short hydration regimen. Nephrotoxicity was assessed in both groups by monitoring serum creatinine levels. A higher nephrotoxicity incidence rate was observed in the conventional regimen group (42.1%, 8 out of 19 patients) compared to the short hydration regimen group, which showed no nephrotoxicity (0%, 0 out of 7 patients), with statistically significant (*p* = 0.039) difference [52]. Likewise, increased AKI cases were observed in only cisplatin-administrated patients (26.2%), while in patients administrated with at least one 5-HT3RA (ondansetron, palonosetron, and granisetron), a low AKI incidence rate (22.6%) was observed [53]. The consistent findings across various studies highlight the need for the vigilant monitoring of renal function in patients undergoing cisplatin therapy, particularly alone. This monitoring is essential not only for the early detection and management of nephrotoxicity, but also for tailoring chemotherapy regimens to minimize renal damage while maximizing therapeutic efficacy. Overall, the outcomes emphasize the importance of balancing the benefits of cisplatin in cancer treatment with its potential to cause renal damage.

The findings of this systematic review and meta-analysis highlight the important clinical implications of monitoring kidney function in cancer patients treated with cisplatin. This is because cisplatin is a widely used chemotherapy agent that is associated with nephrotoxicity. The ability to accurately measure kidney function through GFR, as assessed by ^99^mTc DTPA scans, is important for the timely intervention and management of kidney injury. This monitoring can guide dose adjustments and reduce the risk of AKI, thereby optimizing clinical outcomes and preserving kidney function. Furthermore, the study highlights the need for clinicians to be vigilant in assessing renal parameters regularly in order to identify at-risk patients and implement protective strategies, such as hydration protocols or alternative therapies, to enhance patient safety during cisplatin therapy. Ultimately, these measures can contribute to improved survival rates and quality of life in this vulnerable patient population.

Moreover, certain recommendations should be considered. For instance, regular GFR assessments should be integrated into the treatment plan to identify cisplatin-induced renal impairment promptly. Additionally, baseline and periodic GFR measurements can help tailor cisplatin dosing, minimize nephrotoxicity, and guide decisions regarding supportive therapies such as hydration and the use of nephroprotective agents. Incorporating alternative renal biomarkers alongside ^99^mTc DTPA scans could provide a more comprehensive understanding of renal health in these patients, aiding in the optimization of cancer treatment while mitigating renal risks.

Meanwhile, this study offers several strengths and limitations. A key strength lies in the comprehensive synthesis of data across multiple studies, enhancing the reliability of findings through the aggregation of large sample sizes. This approach provides a robust quantitative assessment of the nephrotoxic effects of cisplatin, which is crucial for informing clinical decisions. Additionally, using ^99^mTc DTPA as a measurement tool facilitates the accurate and sensitive detection of GFR changes, ensuring the precise assessment of renal function. However, the limitations include potential heterogeneity in the included studies, such as variations in patient populations, dosing regimens, and GFR measurement techniques, which may affect the generalizability of the results. Furthermore, the reliance on existing studies means the analysis is subject to the quality and design limitations of those studies, which could introduce bias. Furthermore, due to the unavailability of research papers, very few papers were included in the meta-analysis, which might affect the overall outcomes of the meta-analysis. Future research should investigate alternative therapeutic agents or dosages that can reduce nephrotoxicity without compromising anti-cancer efficacy. In addition, the efficacy of alternative antitumor agents or strategies for hydration strategies during treatment with cisplatin should also be investigated.

## 5. Conclusions

The present study confirms that cisplatin has a significant nephrotoxic effect in cancer patients. The findings show a consistent decrease in GFR across various treatment cycles, emphasizing sensitive GFR measurement as an early indicator of renal injury. These results emphasize the critical need for the rigorous monitoring of renal function during cisplatin therapy to prevent and manage acute kidney injury and chronic nephrotoxicity effectively. Implementing these practices can optimize patient care by balancing the therapeutic benefits of cisplatin with its potential adverse renal effects.

## Figures and Tables

**Table 1:** Summary of general characteristics of the included studies.

Study ID	Country	Study Design	Sample Size	Age (Years)	Gender	Cancer Type	Cisplatin Dosage	Total Cycles	^99^mTc-DTPA Dose
[34]	Denmark	Retrospective	9	23.6	NA	Testicular cancer	20 mg/m^2^	1	300 uCi wasinjected IV
[32]	Pakistan	Descriptive	36	45.3	28:8	Different types of cancer	114.02 mg/m^2^ each cycle	6	5 mCi was injected IV
[33]	Pakistan	Descriptive	33	45	26:7	Solid tumors	114 ± 23 mg/m^2^	6	^99^mTc DTPA was injected IV
[35]	Turkey	Retrospective	26	26.73	26:0	Seminomatous and non-seminomatous testicular carcinoma	20 mg/m^2^ per day for five consecutive days repeated every 21 days	4	^99^mTc DTPA was injected IV
[30]	India	RCT	100	40–60	NA	HNC and CaCx	50 mg/m^2^ IV/weekly	6	^99^mTc DTPA was injected IV
[9]	India	Prospective	64	53	42:22	Locally advanced HNC and uterine CaCx	35 mg/m^2^	6	3 mCi of ^99^mTc DTPA
[36]	Malaysia	Prospective	21	55.1	16:5	Solid tumors	75 mg/m^2^ for each cycle	3	^99^mTc DTPA was injected IV
[31]	India	Retrospective	200	47.86	118:82	GI, HNC, colorectal cancer, thoracic cancer	50–75 mg/m^2^ 31/3–4 week	3	^99^mTc DTPA was injected IV

HNC = head and neck cancer, CaCx = carcinoma cervix, GI = gastrointestinal, RCT = Randomized Controlled Trail, ^99^mTc = Technetium-99m, DTPA = diethyl-triamine-penta-acetic acid, IV = intravenous, NA = not available.

**Table 2:** Summary of outcomes related to cisplatin on GFR and serum creatinine.

Study ID	GFR before Treatment (mL/min/1.73 m^2^)	GFR after Each Cycle (mL/min/1.73 m^2^)	Serum Creatinine mg/dL	AKI Incidence	Conclusion
[34]	NA	1st cycle = 115.7	After treatment = 89.3	0	The degree of chronic nephrotoxicity did not correlate in individual patients with the acute changes in kidney function
[32]	106.74	1st cycle = 95.36, 2nd cycle = 93.54, 3rd cycle = 81.54, 4th cycle = 74.32, 5th cycle = 73.12, 6th cycle = 66.38	NA	NA	CDDP produced an early nephrotoxicity, which manifested in a significant decline in GFR in each cycle
[33]	106.74	1st cycle = 95.36, 2nd cycle = 93.54, 3rd cycle = 81.54, 4th cycle = 74.32, 5th cycle = 73.12, 6th cycle = 66.38	NA	3	GFR is the most sensitive indicator of early cisplatin-induced nephrotoxicity, and PSC1 and Gates method are reliable substitutes for the PSC2 method
[35]	100.23	Immediately after chemotherapy = 86.77After 7 months = 79.7	Baseline = 0.98 immediately after chemotherapy = 1.34, 7 months after chemotherapy = 1.02	NA	Scintigraphic GFR measurement using the Gates method with ^99^mTc-DTPA is a suitable method in the diagnosis of nephrotoxicity occurring due to cisplatin
[30]	HNC = 121, CaCx = 123.7	At the end of 4th week: 80–100	Baseline: HNC = 1.2, CaCx = 0.9, at the end of 4th week: HNC = 1.1–1.5 in >50% cases, CaCx = 0.6–1 in 44% cases	NA	Cisplatin had an impact on renal function
[9]	NA	NA	NA	NA	Baseline RI was detected in 12% more cases when measured by dGFR as compared with SCR level.
[36]	91.17	77.79	NA	3	Utilization of radionuclide methods has shown better detection in GFR changes compared to creatinine-based techniques
[31]	85.49	1st cycle = 66.66, 2nd cycle = 58.09	NA	NA	Nephrotoxicity is a major side effect of platin drugs

NA = not available, HNC = head and neck cancer, CaCx = carcinoma cervix, DTPA = diethyl-triamine-penta-acetic acid, GFR = glomerular filtration rate, PSC = plasma serum creatinine, CDDP = Cis-Diammine-Dichloroplatinum, AKI = acute kidney injury.

**Table 3:** Quality assessment of included studies utilizing Newcastle–Ottawa quality assessment scale (0–9).

Study ID	Selection				Comparability		Outcomes			Score
	Representative Sample	Selection	Ascertainment of Exposure	Outcomes of Interest	Cofounders	Other Factors	Assessment	Follow-Up	Complete Follow-Up	
[34]	*			*		*	*	*	*	6
[32]	*	*		*		*	*	*	*	7
[33]	*	*		*		*	*	*	*	7
[35]	*	*		*		*	*	*	*	7
[9]	*	*		*		*	*	*	*	7
[36]	*	*		*		*	*		*	6
[31]	*	*		*		*	*	*	*	7

Methodological quality assessment of included studies utilizing Newcastle–Ottawa quality assessment scale (0–9). (*) indicates that the study considers one point for each criterion.

## Data Availability

Not applicable.

## Appendix A

**Table A1:** Literature search from different databases.

Databases	Search Terms
PubMed	(“cisplatin”[MeSH Terms] OR “metal-based drug”[Title/Abstract] OR “platinol”[Title/Abstract]) AND (“kidney failure”[Title/Abstract] OR “kidney damage”[Title/Abstract] OR “renal insufficiency”[MeSH Terms] OR “renal failure”[Title/Abstract] OR “kidney dysfunction”[Title/Abstract] OR “nephrotoxicity”[Title/Abstract]) AND (“glomerular filtration rate”[Title/Abstract] OR “GFR”[Title/Abstract] OR “estimated GFR”[Title/Abstract] OR “eGFR”[Title/Abstract]) AND (“diethyl-triamine-penta-acetic acid”[Title/Abstract] OR “DTPA”[Title/Abstract] OR “^99^mTc DTPA”[Title/Abstract] OR “nuclear medicine”[Title/Abstract] OR “renal scintigraphy”[Title/Abstract] OR “creatinine clearance”[Title/Abstract]) AND (“cancer”[Title/Abstract] OR “oncology”[Title/Abstract] OR “neoplasms”[MeSH Terms] OR “malignancy”[Title/Abstract] OR “tumours”[Title/Abstract])
ScienceDirect	(cisplatin OR platinol) AND (kidney failure OR renal failure) AND (glomerular filtration rate OR GFR) AND (cancer OR oncology)
The Cochrane Library	((“cisplatin” OR “metal-based drug” OR “platinol”)):ti,ab,kw AND ((“kidney failure” OR “kidney damage” OR “renal insufficiency” OR “renal failure” OR “kidney dysfunction” OR “nephrotoxicity”)):ti,ab,kw AND ((“glomerular filtration rate” OR “GFR” OR “estimated GFR” OR “eGFR”)):ti,ab,kw AND ((“diethyl-triamine-penta-acetic acid” OR “DTPA” OR “Tc99m DTPA” OR “nuclear medicine” OR “renal scintigraphy” OR “creatinine clearance”)):ti,ab,kw AND ((“cancer” OR “oncology” OR “neoplasms” OR “malignancy” OR “tumours”)):ti,ab,kw
Scopus	(“cisplatin” OR “metal-based drug” OR “platinol”) AND (“kidney failure” OR “kidney damage” OR “renal insufficiency” OR “renal failure” OR “kidney dysfunction” OR “nephrotoxicity”) AND (“glomerular filtration rate” OR “GFR” OR “estimated GFR” OR “eGFR”) AND (“diethyl-triamine-penta-acetic acid” OR “DTPA” OR “Tc99m DTPA” OR “nuclear medicine” OR “renal scintigraphy” OR “creatinine clearance”) AND (“cancer” OR “oncology” OR “neoplasms” OR “malignancy” OR “tumours”)
Google Scholar	(“cisplatin” OR “metal-based drug” OR “platinol”) AND (“kidney failure” OR “kidney damage” OR “renal insufficiency” OR “renal failure”) AND (“glomerular filtration rate” OR “GFR”) AND (“diethyl-triamine-penta-acetic acid” OR “nuclear medicine”) AND (“cancer” OR “oncology” OR “neoplasms”)
